# Prognostic impact of geriatric nutritional risk index on patients with urological cancers: A meta-analysis

**DOI:** 10.3389/fonc.2022.1077792

**Published:** 2023-01-11

**Authors:** Quan Wu, Fagen Ye

**Affiliations:** ^1^ Clinical Laboratory, Huzhou Central Hospital, Affiliated Central Hospital of Huzhou University, Huzhou, Zhejiang, China; ^2^ Department of Urology, Huzhou Central Hospital, Affiliated Central Hospital of Huzhou University, Huzhou, Zhejiang, China

**Keywords:** GNRI, urological cancers, meta-analysis, survival, clinical use

## Abstract

**Background:**

Despite previous research examining the predictive value of the geriatric nutritional risk index (GNRI) in individuals with urological cancers (UCs), results have been conflicting. This study aimed to comprehensively explore the potential link between GNRI and the prognosis of UCs using a meta-analysis.

**Methods:**

The Cochrane Library, PubMed, Embase, and Web of Science databases were systematically and exhaustively searched. We estimated the prognostic importance of the GNRI in patients with UCs by calculating the pooled hazard ratios (HRs) and 95% confidence intervals (CIs) on survival outcomes. Publication bias was identified using Egger’s test and Begg’s funnel plot.

**Results:**

Eight trials with 6,792 patients were included in our meta-analysis. Patients with UCs who had a lower GNRI before treatment had a higher risk of experiencing worse overall survival (HR = 2.62, 95% CI = 1.69–4.09, p < 0.001), recurrence-free survival/progression-free survival (HR = 1.77, 95% CI = 1.51–2.08, p < 0.001), and cancer-specific survival (HR = 2.32, 95% CI = 1.28–4.20, p = 0.006). Moreover, the subgroup analysis did not change the predictive significance of the GNRI in individuals with UCs. Neither Egger’s nor Begg’s test indicated substantial bias in this analysis.

**Conclusion:**

As a result of our meta-analysis, we found that a low GNRI strongly predicts poor prognosis for patients with UCs. A lower pretreatment GNRI indicates poor survival outcomes in UCs.

## 1 Introduction

Urological cancers (UCs), including urothelial carcinoma (UC), renal cell carcinoma (RCC), and prostate cancer (PCa), are the primary causes of public health issues globally (1). UCs account for 380,480 new cases and 46,620 cancer-related deaths in men in the United States by 2022 (2). The incidence and mortality of UCs have been increasing in recent years, and UCs are more prevalent in Western countries than in Eastern regions (3, 4). Personalized medicine plays an important role in the treatment of UCs. The foundation of medical care includes androgen deprivation therapy (ADT) for PCa, tyrosine kinase inhibitors for RCC, and cytotoxic chemotherapy for UC (5). Patients undergoing urological oncology surgeries, such as radical prostatectomy, radical cystectomy, and radical nephroureterectomy, show a particular community at risk of poor prognosis (6). For example, for patients with bladder who underwent radical cystectomy (RC), the overall 3, 5 and 10-year survival after RC was 62%, 52% and 37%, respectively (6). However, finding new prognostic markers for patients with UCs is crucial for the design of therapeutic approaches.

Numerous studies have demonstrated a robust association between malnutrition and poor prognosis in patients with cancer. Nutritional evaluations, such as the prognostic nutritional index (7), controlling nutritional status score (8), and geriatric nutritional risk index (GNRI) (9), are commonly used to evaluate malnutrition in patients (7–9). In 2005, Bouillanne et al. (10) initially suggested the GNRI to evaluate the likelihood of death or disability in medically stable older adult individuals. The ideal weight, current weight, and serum albumin level (10) were used to determine GNRI. GNRI was calculated as GNRI = 14.89 * albumin (mg/dl) + 41.7 * (current/ideal body) weight. Nutritional status in patients with cancer may be evaluated using the GNRI because it is a straightforward method. Previous research has revealed the predictive usefulness of the GNRI in many different forms of cancer, including gastric cancer (11), hepatocellular carcinoma (9), pancreatic cancer (12), and oral squamous cell carcinoma (13). The prognostic factor of GNRI in patients with UC has been the subject of several studies with varying results (14–21). We collated relevant literature and conducted this study to evaluate the correlation between prognosis and GNRI in patients.

## 2 Materials and methods

### 2.1 Ethics statement

This meta-analysis did not require the use of an institutional review board or ethical committee. Additionally, the primary data were obtained from previously published research; therefore, there was no direct effect on the participants.

### 2.2 Study guideline

The Preferred Reporting Items for Systematic Reviews and Meta-Analyses guidelines were used to compile the data for this meta-analysis (22).

### 2.3 Literature search

We systematically and extensively searched the Cochrane Library, Embase, PubMed, and Web of Science databases. Our exhaustive and targeted search methodology consisted of the following steps: (geriatric nutritional risk index OR GNRI) AND (bladder cancer OR renal cell cancer OR prostate cancer OR urothelial cancer OR urological cancer OR urinary cancer). A new search update was implemented on September 10, 2022. Articles written in languages other than English were also excluded. Furthermore, we also analyzed all the cited sources of the reviews and studies to find other papers that were relevant to our topic.

### 2.4 Inclusion and exclusion criteria

The inclusion criteria were as follows: (i) patients with upper tract urothelial cancer, bladder cancer, PCa, RCC, and UC were pathologically diagnosed; (ii) patients were divided into subgroups based on their GNRI; (iii) a GNRI cut-off value was determined; (iv) the GNRI was calculated as 14.89 × albumin (mg/dl) + 41.7 × (present/ideal body) weight (kg) before treatment; (v) hazard ratios (HRs) and 95% confidence intervals (CIs) were reported or adequate data were provided to compute them; and (vi) recurrence-free survival (RFS), cancer-specific survival (CSS), overall survival (OS), and progression-free survival (PFS) were reported. The following studies were excluded: animal studies, studies that did not provide enough data for analysis, studies that were duplicated and featured the same patients, reviews and conference abstracts, letters and case reports, and comments.

### 2.5 Data extraction and quality assessment

The literature review was conducted by two scholars working separately (QW and FY). All disagreements were discussed and resolved verbally until agreement was reached. Data from relevant studies included the first author’s name, year of publication, sample size, country, sex, time period, type of cancer, study design, study center (multicenter or single-center), treatment, tumor-node-metastasis (TNM) stage, duration of follow-up, GNRI cut-off value, type of survival analysis, survival outcomes, and HRs and 95% CIs. When both multivariate and univariate HRs and 95% CIs were used, the results of the multivariate analysis (MVA) were employed. In cases where only UVA was available, the HRs and 95% CIs were used instead. Each study included in the list was scored on the Newcastle-Ottawa scale (NOS) (23) to evaluate the research design quality. The final NOS score may range from 0 to 9, with points awarded for comparability (1–2), patient selection (0–4), and outcome (0–3). A high-quality study received a score of ≥ 6.

### 2.6 Statistical analysis

The predictive significance of the GNRI in patients with UCs was evaluated by calculating the 95% CI and HR for survival outcomes. The I^2^ statistic and Cochrane Q statistic were used to assess statistical heterogeneity between studies. Owing to the low levels of heterogeneity, indicated by an I^2^ value below 50% and a Q-test significance level above 0.10, a fixed-effects model (FEM) was used. Without this information, a random-effects model (REM) was utilized. To determine the origin of the observed variation, a subgroup analysis was performed, stratified by several clinicopathological characteristics. Publication bias was determined using Egger’s test and Begg’s funnel plot. The Stata version 12.0 was used for all statistical analysis (Stata Corporation, College Station, TX, USA) was used for all statistical analyses. Statistical significance was set at p < 0.05.

## 3 Results

### 3.1 Study selection

**Figure 1:**
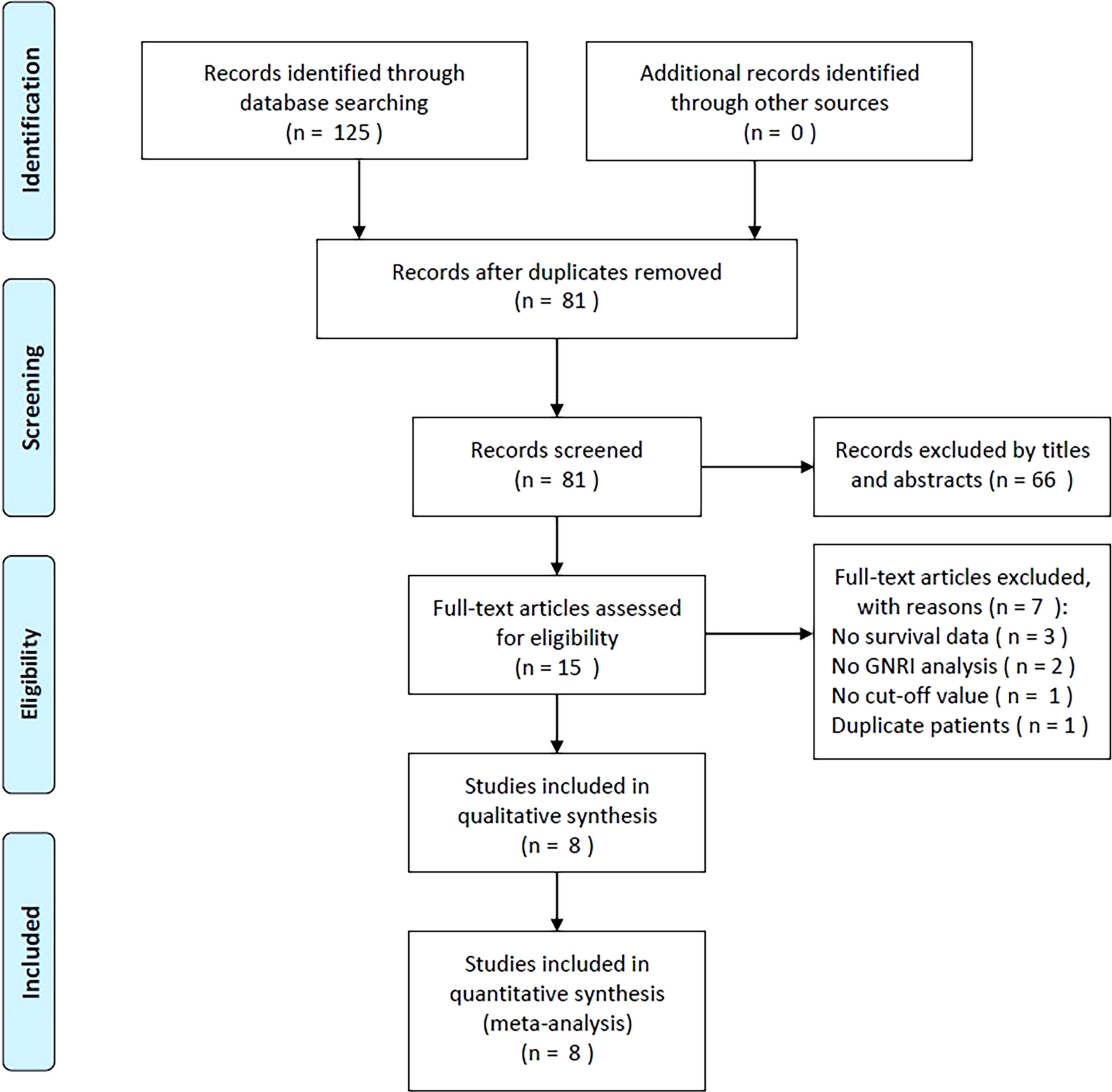
The flow diagram of this meta-analysis.

As shown in **Figure 1**, the initial literature search generated a total of 125 items. After filtering out 44 duplicates, the abstracts and titles of 81 papers were read. Thereafter, 66 papers were discarded, leaving only 15 for the full-text analysis. Seven studies were excluded for the following reasons: (1) they did not provide survival data (n = 3), (2) they did not perform a GNRI analysis (n = 2), (3) they did not determine a GNRI cut-off value (n = 1), and (4) they included patients who had already been studied (n = 1). Eight studies with 6,792 patients (14–21) were included in the final meta-analysis (**Figure 1**).

### 3.2 Features of the included research

**Table 1** shows the typical characteristics of the included studies. The articles considered were published in full-text format in the English language between 2015 and 2022 (14–21). Four studies were performed in Japan (15, 16, 19, 21), two in China (14, 20), and one each in Korea (17) and Taiwan (18). The sample sizes ranged from 68 to 4,591, with a median of 319.5. Four studies recruited patients with RCC (14, 15, 17, 20), two studies enrolled patients with PCa (16, 18), and two studies included patients with UC (19, 21). Seven studies were retrospective studies (15–21) and one was a prospective trial (14). Five studies recruited patients with TNM stage IV (14, 16, 18, 19, 21) and three studies enrolled patients with TNM stages I–III (15, 17, 20). Three studies included patients receiving surgery (15, 17, 20), two studies recruited patients undergoing chemotherapy (18, 19), and one study used ADT (16), immune checkpoint inhibitor (21), and targeted therapy (14). Seven studies adopted 92 as the cut-off value for the GNRI (14, 16–21) and one study adopted 98 (15). The significance of the GNRI as an OS prognostic factor was revealed in six studies (14, 16, 18–21), three studies presented the association between the GNRI and RFS (15, 17, 20), two studies reported the HR and 95%CI for PFS (18, 19), and three studies demonstrated a correlation between the GNRI and CSS (15–17). Six studies described the HRs and 95% CIs from the MVA (14, 17–21), and two studies reported data from the UVA (15, 16). Five studies were multicenter (14, 16, 17, 19, 21) and three were single-center (15, 18, 20). The NOS score of the considered studies varied from 7 to 9, with a median of 8, showing that the methodology of all considered studies was of a high standard.

**Table 1:** Basic characteristics of included in this meta-analysis.

Author	Year	Country/ region	Sample size	Age (years) Median(range)	Cancer type	Gender (M/F)	Study duration	Study design	Study center	TNM stage	Treatment	Follow-up (month) Median(range)	Cut-off value of GNRI	Survival outcomes	Survival analysis type	NOS score
Gu, W.	2015	China	300	56.2(27-81)	RCC	203/97	2009-2013	Prospective	Multicenter	IV	Targeted therapy	30.8	92	OS	MVA	8
Miyake, H.	2017	Japan	432	≤70: 164 >70: 268	RCC	277/155	2005-2011	Retrospective	Single center	I-III	Surgical resection	1-100	98	RFS, CSS	UVA	7
Okamoto, T.	2019	Japan	339	72	PCa	339/0	2005-2017	Retrospective	Multicenter	IV	ADT	26(12-53)	92	OS, CSS	UVA	8
Kang, H. W.	2020	Korea	4,591	61	RCC	3,367/1,224	1988-2015	Retrospective	Multicenter	I-III	Surgical resection	37	92	RFS, CSS	MVA	9
Chang, L. W.	2021	Taiwan	170	74	PCa	170/0	2006-2012	Retrospective	Single center	IV	Chemotherapy	22.49(11.35-41.32)	92	OS, PFS	MVA	8
Naiki, T.	2021	Japan	68	71(49-87)	Urothelial carcinoma	55/13	2016-2020	Retrospective	Multicenter	IV	Chemotherapy	12.9(1.7-50.2)	92	OS, PFS	MVA	8
Tang, Y.	2021	China	694	≤60: 449 >60: 245	RCC	442/252	2009-2014	Retrospective	Single center	I-III	Surgical resection	60.9	92	OS, RFS	MVA	7
Isobe, T.	2022	Japan	198	70(37-85)	Urothelial carcinoma	163/35	2009-2021	Retrospective	Multicenter	IV	ICI	1-60	92	OS	MVA	8

GNRI, Geriatric Nutrition Risk Index; RCC, renal cell carcinoma; PCa, prostate cancer; UC, urothelial carcinoma; OS, overall survival; CSS, cancer-specific survival; RFS, recurrence-free survival; PFS, progression-free survival; ADT, androgen-deprivation therapy; ICI, immune checkpoint inhibitor; MVA, multivariate analysis; UVA, univariate analysis; TNM, tumor-node-metastasis; NOS, Newcastle-Ottawa Scale; M, male; F, female.

### 3.3 GNRI and OS in UCs

**Figure 2:**
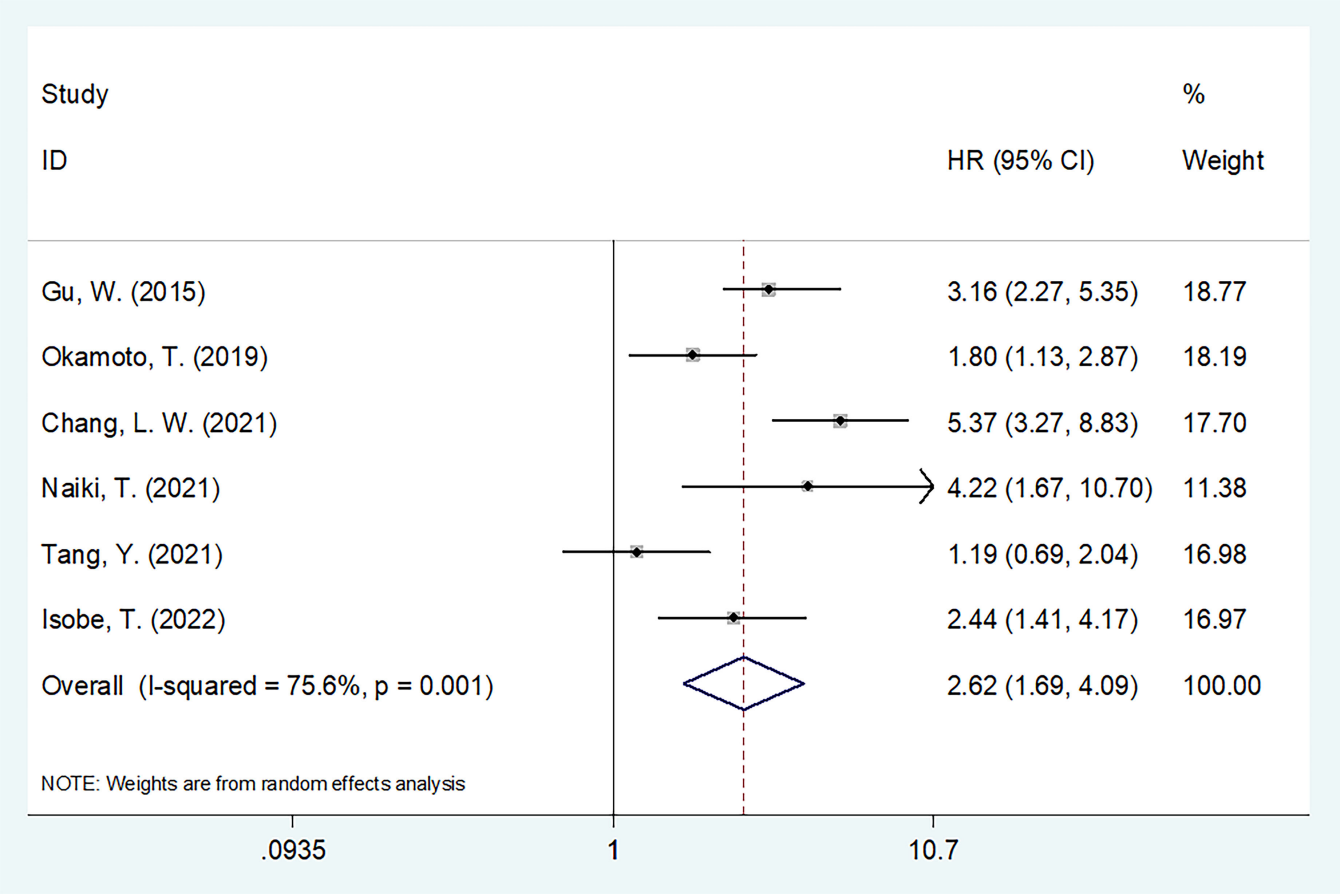
The forest plot of the association of pretreatment GNRI with overall survival (OS) of patients with UCs.

The predictive importance of the GNRI for OS in patients with UCs was revealed in six investigations, including a total of 1,769 participants (14, 16, 18–21). In this case, substantial heterogeneity (I^2^=75.6%, Ph=0.001) necessitated REM deployment. As shown in **Table 2** and **Figure 2**, the combined results indicated that a low GNRI was significantly associated with poor OS in patients with UCs (HR = 2.62, 95% CI = 1.69–4.09, p < 0.001). The subgroup analysis revealed that regardless of study design, type of survival analysis, or sample size, a low GNRI was a clear indication of worse OS (**Table 2**). Patients with UC and PCa, but not RCC, had a low GNRI and poor OS (**Table 2**).

**Table 2:** Subgroup analysis of the prognostic value of GNRI for OS in patients with urologic cancers.

Factors	No. of studies	No. of patients	Effects model	HR (95%CI)	p	Heterogeneity I^2^ (%) Ph	
Overall	6	1,769	REM	2.62 (1.69-4.09)	<0.001	75.6	0.001
Sample size							
≤300	4	736	FEM	3.54 (2.71-4.62)	<0.001	39.1	0.177
>300	2	1,033	FEM	1.51 (1.06-2.15)	0.022	22.3	0.257
Cancer type							
RCC	2	994	REM	1.97 (0.76-5.12)	0.165	87.0	0.006
PCa	2	509	REM	3.10 (1.06-9.04)	0.039	89.9	0.002
UC	2	266	FEM	2.80 (1.76-4.48)	<0.001	0	0.318
Study design							
Retrospective	5	1,469	REM	2.53 (1.45-4.41)	0.001	79.4	0.001
Prospective	1	300	–	3.16 (2.06-4.84)	<0.001	–	–
Study center							
Multicenter	4	905	FEM	2.55 (1.96-3.31)	<0.001	29.5	0.235
Single center	2	864	REM	2.54 (0.58-11.11)	0.216	93.8	<0.001
TNM stage							
I-III	1	694	–	1.19 (0.69-2.05)	0.529	–	–
IV	5	1,075	REM	3.06 (2.06-4.57)	<0.001	63.7	0.026
Survival analysis							
MVA	5	1,430	REM	2.86 (1.70-4.80)	<0.001	77.1	0.002
UVA	1	339	–	1.80 (1.13-2.87)	0.013	–	–

REM, random-effects model; FEM, fixed-effects model; RCC, renal cell carcinoma; PCa, prostate cancer; UC, urothelial carcinoma; MVA, multivariate analysis; UVA, univariate analysis.

### 3.4 GNRI and RFS/PFS in UCs

**Figure 3:**
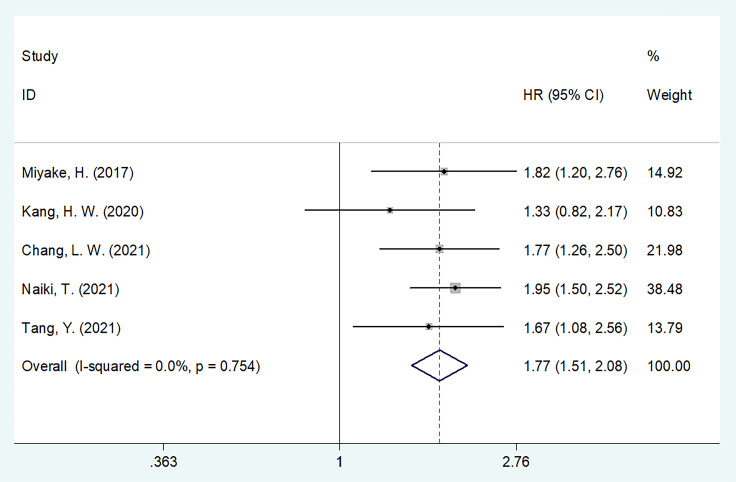
The forest plot of the association of pretreatment GNRI with recurrence-free survival/progression-free survival (RFS/PFS) of patients with UCs.

We merged RFS and PFS into the RFS/PFS groups because they were both event-free survival endpoints. Five studies comprising 5,955 patients (15, 17–20) reported the relationship between RFS/PFS and GNRI. The pooled HR and 95% CI were as follows: p < 0.001, HR = 1.77, 95% CI = 1.51–2.08 in the FEM (**Figure 3**, **Table 3**), which suggested that patients with UCs with low GNRI had poor RFS/PFS. The prognostic significance of GNRI for RFS/PFS remained significant in various subgroups of sample size, cancer type, study center, TNM stage, and cut-off value, as shown in **Table 3** from the subgroup analysis.

**Table 3:** Subgroup analysis of the prognostic value of GNRI for RFS/PFS in patients with urologic cancers.

Factors	No. of studies	No. of patients	Effects model	HR (95%CI)	p	Heterogeneity I^2^ (%) Ph	
Overall	5	5,955	FEM	1.77 (1.51-2.08)	<0.001	0	0.754
Sample size							
≤300	2	238	FEM	1.88 (1.53-2.31)	<0.001	0	0.676
>300	3	5,717	FEM	1.62 (1.26-2.09)	<0.001	0	0.626
Cancer type							
RCC	3	5,717	FEM	1.62 (1.26-2.09)	<0.001	0	0.626
PCa	1	170	–	1.77 (1.26-2.50)	0.001	–	–
UTC	1	68	–	1.95 (1.50-2.52)	<0.001	–	–
Study center							
Multicenter	2	4,659	FEM	1.79 (1.42-2.25)	<0.001	44.6	0.179
Single center	3	1,296	FEM	1.76 (1.40-2.20)	<0.001	0	0.959
TNM stage							
I-III	3	5,717	FEM	1.62 (1.26-2.09)	<0.001	0	0.626
IV	2	238	FEM	1.88 (1.53-2.31)	<0.001	0	0.676
Cut-off value							
92	4	5,523	FEM	1.77 (1.48-2.10)	<0.001	0	0.597
98	1	432	–	1.82 (1.20-2.76)	0.005	–	–
Survival analysis							
MVA	4	5,523	FEM	1.77 (1.48-2.10)	<0.001	0	0.597
UVA	1	432	–	1.33 (0.82-2.17)	0.247	–	–

REM, random-effects model; FEM, fixed-effects model; RCC, renal cell carcinoma; PCa, prostate cancer; UC, urothelial carcinoma; MVA, multivariate analysis; UVA, univariate analysis.

### 3.5 GNRI and CSS in UCs

**Figure 4:**
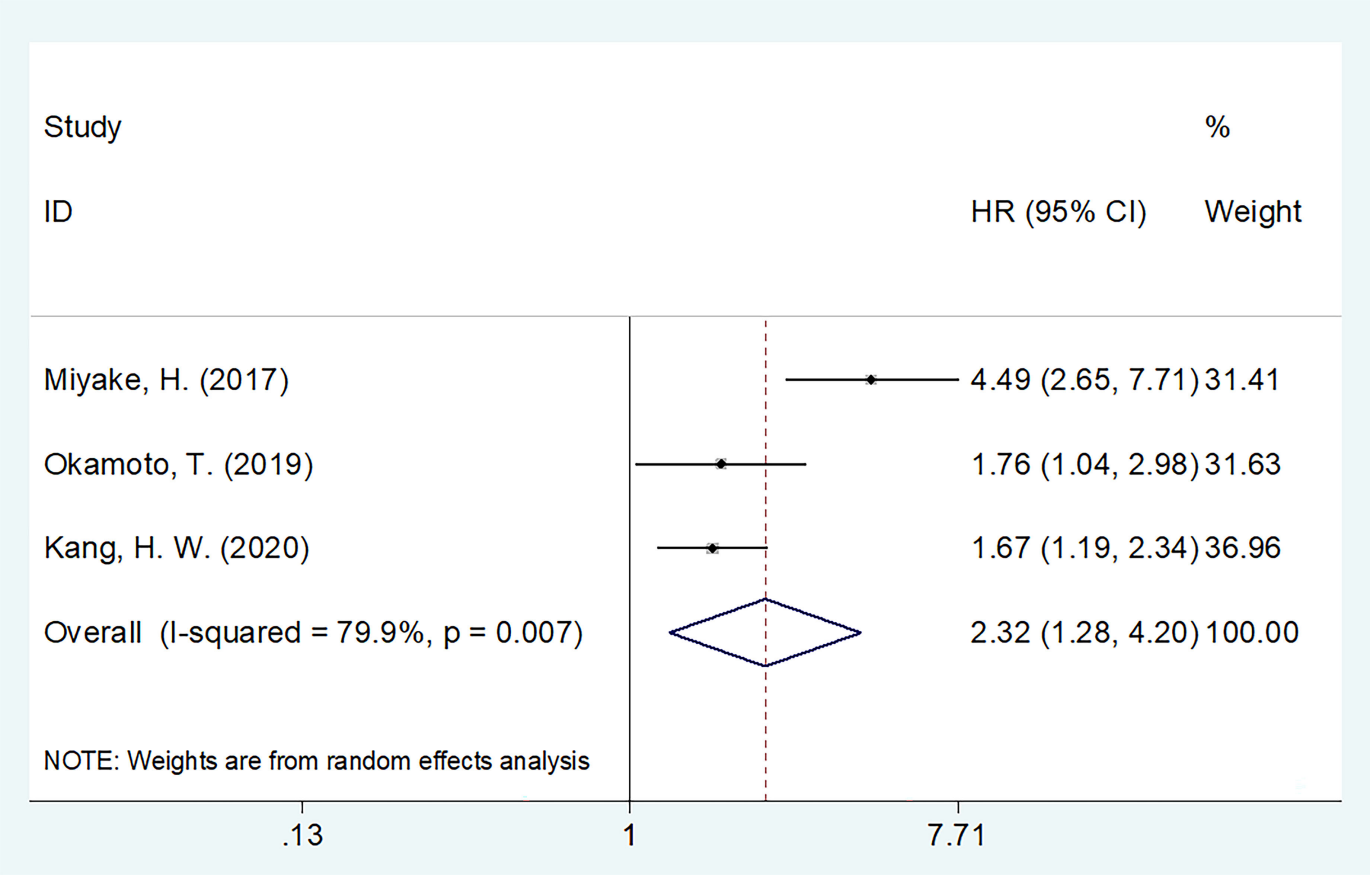
The forest plot of the association of pretreatment GNRI with cancer-specific survival (CSS) of patients with UCs.

Three studies, consisting of 5,362 patients (15–17) described the HRs and 95% CIs for CSS. REM was used, and the combined outcomes were as follows: HR = 2.32, 95% CI = 1.28–4.20, p = 0.006 (**Figure 4**). As shown in **Table 4**, subgroup analysis revealed that decreased GNRI was an important prognostic marker for poor CSS, regardless of the study center and cut-off value in patients with UCs.

**Table 4:** Subgroup analysis of the prognostic value of GNRI for CSS in patients with urologic cancers.

Factors	No. of studies	No. of patients	Effects model	HR (95%CI)	p	Heterogeneity I^2^ (%) Ph	
Overall	3	5,362	REM	2.32 (1.28-4.20)	0.006	79.9	0.007
Cancer type							
RCC	2	5,023	REM	2.68 (1.02-7.05)	0.046	89.4	0.002
PCa	1	339	–	1.76 (1.04-2.98)	0.035	–	–
Study center							
Multicenter	2	4,930	FEM	1.70 (1.28-2.26)	<0.001	0	0.870
Single center	1	432	–	4.49 (2.63-7.66)	<0.001	–	–
TNM stage							
I-III	2	5,023	REM	2.68 (1.02-7.05)	0.046	89.4	0.002
IV	1	339	–	1.76 (1.04-2.98)	0.035	–	–
Cut-off value							
98	1	432	–	4.49 (2.63-7.66)	<0.001	–	–
92	2	4,930	FEM	1.70 (1.28-2.26)	<0.001	0	0.870
Survival analysis							
MVA	1	4,591	–	1.67 (1.19-2.34)	0.003	–	–
UVA	2	771	REM	2.81 (1.12-7.03)	0.027	83.3	0.014

REM, random-effects model; FEM, fixed-effects model; RCC, renal cell carcinoma; PCa, prostate cancer; MVA, multivariate analysis; UVA, univariate analysis.

### 3.6 Publication bias

**Figure 5:**
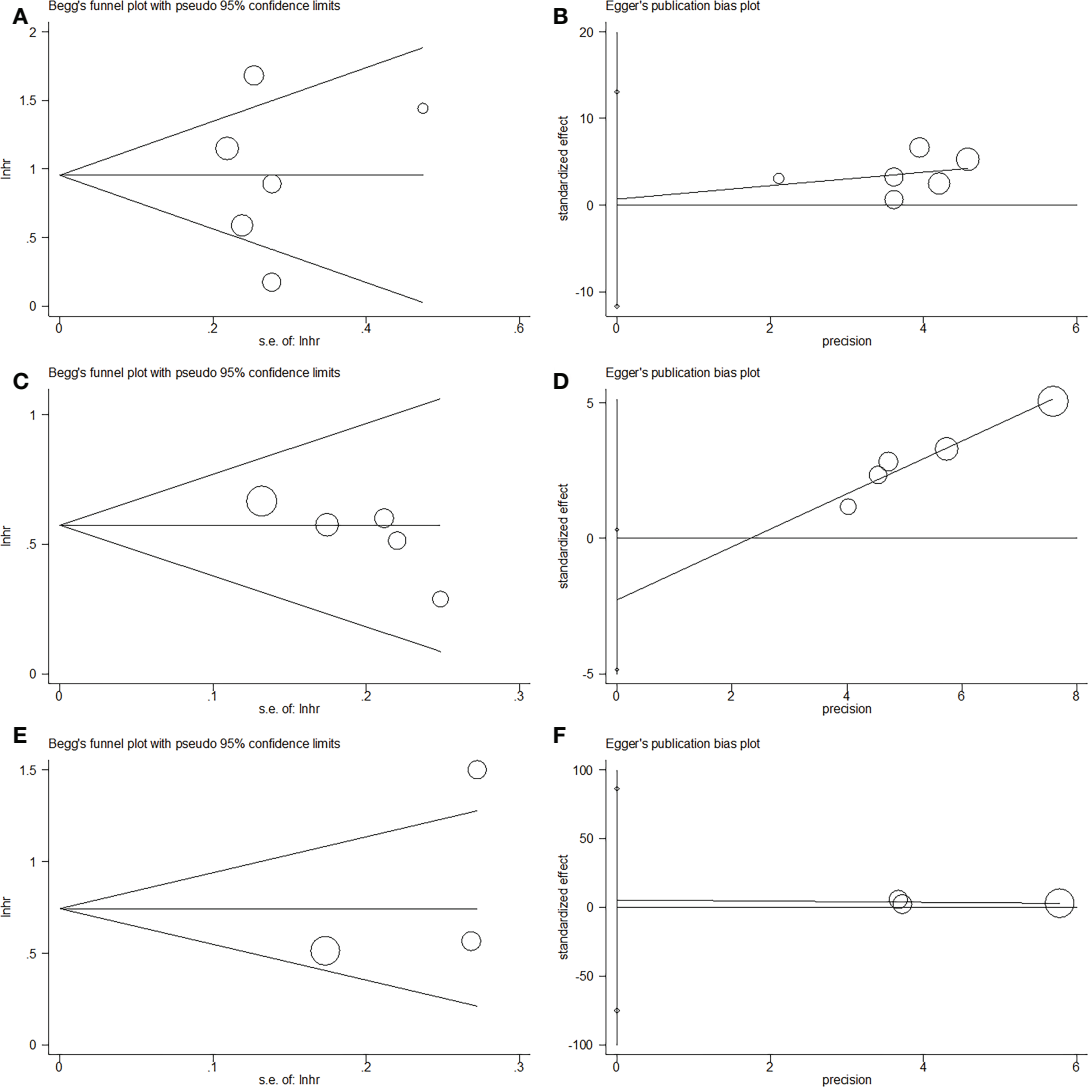
Publication bias by Begg’s test and Egger’s test in this meta-analysis. **(A)** Begg’s test for OS, p=0.851; **(B)** Egger’s test for OS, p=0.883; **(C)** Begg’s test for RFS/PFS, p=0.086; **(D)** Egger’s test for RFS/PFS, p=0.068; **(E)** Begg’s test for CSS, p=0.296; **(F)** Egger’s test for CSS, p=0.548.

This meta-analysis did not exhibit any significant publication bias according to Egger’s test and Begg’s test (**Figure 5**).

## 4 Discussion

Prior research has shown conflicting results regarding the prognostic efficacy of GNRI in patients with UCs. In the present meta-analysis, we included eight studies with a total of 6,792 patients and found that low GNRI predicted poor RFS/PFS, CSS, and OS in patients with UCs. In addition, the prognostic impact of the GNRI in these patients remained stable in diverse subgroups. The publication bias test identified non-significant publication bias and validated the accuracy of our findings. To our knowledge, this is the first meta-analysis to explore the association between pre-treatment survival outcomes and GNRI in UCs. Based on our meta-analysis, we know that a low GNRI is an easy and reliable prognostic indicator for patients with UCs in clinical practice.

The GNRI is a nutritional index based on body weight and albumin level. Therefore, the roles of these two components in cancer can provide insights into the processes underlying the association between GNRI and prognosis in UCs. Albumin levels are often used to assess patients’ nutritional and inflammatory health when dealing with UCs. There was a correlation between low albumin levels and increased fetoprotein levels, portal vein thrombosis, larger maximal tumor diameters, increased tumor multifocality, and shorter overall survival time (24). Therefore, a lower serum albumin level directly indicates the malnutrition status of patients with cancer. Moreover, current evidence shows that malnutrition is a common issue among patients with cancer, with an incidence of 39–71% (25, 26). Researchers have found that low albumin levels are a strong predictor of poor health outcomes in patients with advanced cancer (27). In contrast, weight is a proxy for the extent of a systemic ailment and reserves of protein and calories. To calculate the GNRI, we must first calculate the body mass index by comparing an individual’s actual weight to their ideal weight. It is well established that low body mass index is associated with poor prognosis in patients with cancer (28).

Some recent studies have provided pivotal evidence for the clinical use of nutritional indices for the prognosis of patients with urological cancers (29, 30). A recent single-center retrospective study including 510 cases showed that the fibrinogen-to-albumin ratio (FAR) in patients with bladder cancer who had elevated preoperative FAR might be more likely to have advanced-stage cancer and malignancy (29). Another recent study proposed that the lymphocyte-to-monocyte ratio could be a promising prognostic indicator for tumor progression in patients with bladder cancer (30).

Several recent meta-analyses have documented the prognostic importance of GNRI (31–34). In a meta-analysis of 11 trials, Zhou et al. demonstrated that a low GNRI was associated with poor CSS and OS in patients with esophageal cancer (31). In a meta-analysis of 3,239 patients, Xu et al. found that a low GNRI score was associated with a higher risk of death and postoperative complications in Asian patients with colon cancer (35). The authors of a recent meta-analysis of 8 studies conducted by Wang et al. (36) found that low GNRI levels were associated with shorter RFS, CSS, and OS in patients with lung cancer. Consistent with earlier findings in other cancer types, our meta-analysis showed that a lower GNRI was an effective prognostic predictor of RFS/PFS, CSS, and OS in patients with UC.

This meta-analysis has some limitations. First, all included studies were conducted in East Asia. Therefore, it is important to confirm our meta-analysis results in locations other than Asia. Second, because many studies in this meta-analysis were retrospective, there is a possibility of intrinsic selection bias and heterogeneity. Third, there was no consistent GNRI cut-off value across studies that were considered; hence, an ideal cut-off value should be determined. It is important to conduct multinational large-scale prospective trials across nations to corroborate our findings.

In summary, our meta-analysis concluded that a low GNRI significantly predicts worse outcomes for patients with UC. A lower pretreatment GNRI indicates poor survival outcomes in UCs. The GNRI may be a potential parameter for evaluating prognosis and developing appropriate treatment approaches for patients with UC.

## Data availability statement

The original contributions presented in the study are included in the article/supplementary material. Further inquiries can be directed to the corresponding author.

## Author contributions

QW and FY designed the study. QW and FY established the process of literature selection and screened the abstracts and articles. QW analyzed data and wrote the main manuscript. All authors reviewed and approved the final manuscript.
